# Purine Nucleoside Phosphorylase Deficiency in Two Unrelated Patients with Autoimmune Hemolytic Anemia and Eosinophilia: Two Novel Mutations

**DOI:** 10.34172/aim.2023.105

**Published:** 2023-12-01

**Authors:** Zahra Alizadeh, Mohsen Badalzadeh, Hanieh Heydarlou, Leila Shakerian, Maryam Mahlooji rad, Fariborz Zandieh, Mohammad Reza Fazlollahi

**Affiliations:** ^1^Immunology, Asthma and Allergy Research Institute, Tehran University of Medical Sciences, Tehran, Iran; ^2^Children’s Medical Center, Pediatrics Center of Excellence, Tehran University of Medical Sciences, Tehran, Iran; ^3^Department of Asthma, Allergy and Immunology, Bahrami Children Hospital, Tehran University of Medical Sciences, Tehran, Iran

**Keywords:** Immunodeficiency, Novel mutations, Purine nucleoside phosphorylase, Purine nucleoside phosphorylase deficiency

## Abstract

Two Iranian patients with purine nucleoside phosphorylase (PNP) deficiency are described in terms of their clinical and molecular evaluations. PNP deficiency is a rare form of combined immunodeficiency with a profound cellular defect. Patients with PNP deficiency suffer from variable recurrent infections, hypouricemia, and neurological manifestations. Furthermore, patient 1 developed mild cortical atrophy, and patient 2 presented developmental delay, general muscular hypotonia, and food allergy. The two unrelated patients with developed autoimmune hemolytic anemia and T cells lymphopenia and eosinophilia were referred to Immunology, Asthma and Allergy Research Institute (IAARI) in 2019. After taking blood and DNA extraction, genetic analysis of patient 1 was performed by PCR and direct sequencing and whole exome sequencing was applied for patient 2 and the result was confirmed by direct sequencing in the patient and his parents. The genetic result showed two novel variants in exon 3 (c.246_285+9del) and exon 5 (c.569G>T) *PNP* (NM_000270.4) in the patients, respectively. These variants are considered likely pathogenic based on the American College of Medical Genetics and Genomics (ACMG) guideline. PNP deficiency has a poor prognosis; therefore, early diagnosis would be vital to receive hematopoietic stem cell transplantation (HSCT) as a prominent and successful treatment.

## Introduction

Combined immunodeficiency disease caused by purine nucleoside phosphorylase (PNP) deficiency (OMIM# 613179) is a rare autosomal recessive genetic disorder that severely impairs the function of T cells. Despite this, normal B cell function and number have been reported.^1^ Patients with PNP deficiency usually suffer from severe infections, failure to thrive, hypouricemia, malignancies, and neutropenia. Neurologic manifestations are present in about two-thirds of the patients and include hypotonia, developmental delay, mental retardation, and ataxia.^2^ One-third of these patients are diagnosed with autoimmune hemolytic anemia and immune thrombocytopenia.^3^

PNP deficiency has been reported in about 4% of the patients with severe T cell deficiencies.^4^ PNP is known as an essential enzyme in the purine salvage pathway that reversibly converts inosine to hypoxanthine and guanosine to guanine.^5^ PNP-deficient patients have high levels of deoxyguanosine, guanosine and decreased concentrations of guanine and uric acid in their plasma and urine.^6^ Deoxyguanosine triphosphate (dGTP) accumulation in the mitochondria is toxic to T lymphocytes, inhibits mitochondrial DNA repair, and increases T lymphocyte apoptosis during the selection in thymus.^7,8^ The neurological dysfunction experienced by patients is caused by the insufficient presence of dGTP in brain tissue, as well as the accumulation of purines within the brain.^9^

 Here, we present the clinical and immunological findings of two patients with PNP deficiency who were referred to the Immunology, Asthma and Allergy Research Institute (IAARI). They presented novel features of the disease and the result of this study may provide valuable evidence for early diagnosis.

## Case Report

 The first patient was a 20-month-old male and the third child of non-consanguineous parents referred to IAARI with recurrent respiratory tract infections, chronic diarrhea, rectal prolapse, and gastroesophageal reflux since infancy. He had a five-time history of hospital admissions since he was 15 months old for gastroenteritis, sepsis and severe anemia. The patient was regularly immunized with all standard vaccines including BCG and OPV-1 as live vaccinations without any complications.

**Figure 1:**
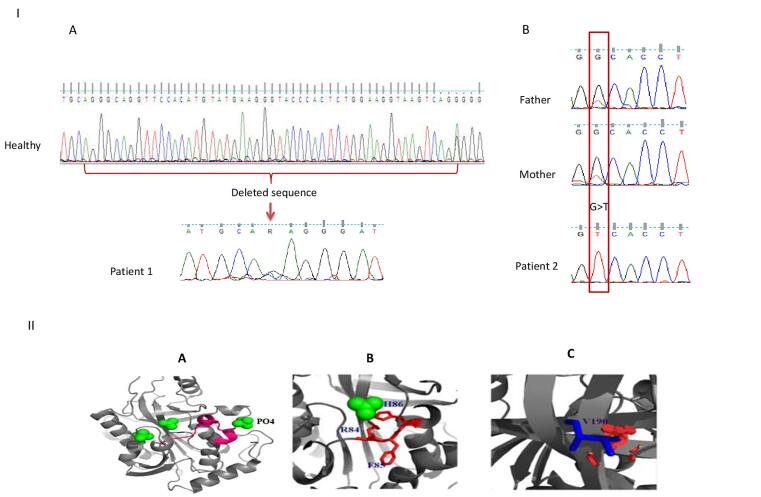
PNP Gene Mutations and Ribbon Diagram of PNP Monomer. I) Schematic presentation of PNP gene mutations in patients. Deleted sequences (c.246_285 + 9del) in patient 1 compared to healthy control (A). Homozygote c.569G > T mutation in patient 2 and heterozygote mutation in his parents (B). II) Ribbon diagram for the monomer of PNP by PYMOL. The crystal structure at 2.3 Å resolution; PDB 1M73 was used as the model structure for the analysis. Structure visualization and mutagenesis analysis were performed with the PyMOL program (DeLano Scientific, San Carlos, CA, USA). Deletion of p. Gly83_Lys95 in patient 2 is shown in pink (A). The phosphate and sulfate binding site is deleted in patient 1 (B). Substitution of Gly to Val in patient 2 (C)

He had developmental delay and his brain CT scan revealed mild cortical atrophy at one year of age. Diminished granulopoiesis in different myeloid precursors with mature granulocytes and a reduced number of erythroid precursors were shown in his bone marrow aspirate (BMA). Laboratory investigations showed Coombs-positive hemolytic anemia treated with several times blood transfusions. His immunological workup showed eosinophilia, lymphopenia, and low T cell numbers and function (Table 1). Biochemical evaluation revealed undetectable uric acid in his serum sample (Table 1). Based on his severe T cell lymphopenia, low uric acid and clinical findings, PNP deficiency was the most probable diagnosis. The genomic DNA of the patient was investigated for *PNP* gene mutation by PCR (using specific primers and an Amplicon PCR kit) and the product was subjected to Sanger sequencing (ABI 3730XL genetic analyzer, Applied Biosystems). The sequencing results were analyzed by the FinchTV program. A novel large homozygous deletion (c.246_285 + 9del, p. Gly83_Lys95 + 9 bp intron) was found in exon 3 of the *PNP* gene (NM_000270.4) (Figure 1A). This variant has been indicated as likely pathogenic based on VarSome tools.

**Table 1:** Immunological and Biomedical Evaluations of two Patients with PNP Deficiency

**Lab tests**	**Patient 1** **values at 2 years (Normal range)**^a^	**Patient 2** **values at 6 years (Normal range)**^a^
Hb (g/dL)	8.8 (12.0 (10.5))	10.7 (12.5 (11.5))
WBC (10^3^/µL)	5.49 (10.6 (6-17))	2.94 (8.5 (5-15.5))
NEUT (10^3^/µL)	3.96 (1.5-8.5)	1.29 (1.5-8)
LYMPH (10^3^/µL)	0.34 (3-9.5)	0.26 (1.5-7)
MONO (10^3^/µL)	0.98 (0.5 mean)	0.41 (0.4 mean)
EOS (10^3^/µL)	0.42 (0.04-0.4)	1 (0.01-0.2)
CD3 count (%)	14 (53-73)	42.2 (60-79)
CD4 count (%)	13 (32-51)	25.7 (31-47)
CD8 count (%)	9 (14-39)	12.9 (18-35)
CD 19 count (%)	14 (16-35)	7.74 (16-35)
CD 16/56 count (n)*	0.071 (0.18-0.92)	0.14 (0.10-0.48)
IgE (Iu/mL)	0.6 (0.31-29.5)	160 (1.02-161.3)
IgM (mg/dL)	223 (48-168)	87 (48-207)
IgG (mg/dL)	1863 (428-1051)	1289 (633-1280)
IgA (mg/dL)	63 (14-123)	132 (33-202)
Isohemagglutinin titers	1/32 (More than 1/8)	1/2 (More than 1/8)
DTH to PPD antigen	Negative	-
DTH to DT antigen	Negative	-
TREC (copies/3.2 mmDBS)	0	-
Uric acid (mg/dl)	undetectable (2.4-6.8)	1 (2.4-5.9)

^a^ Age-Related Reference Range from The Harriet Lane Handbook, Jason W. Custer and Rachel E. Rau, Eighteenth Edition.

 The patient died because of a respiratory infection at 20 months of age. Unfortunately, the samples of the patient’s parents were not available.

The second patient was a six-year-old boy from consanguineous parents referred to IAARI due to recurrent infections. His physical examination showed that he suffered from general muscular hypotonia, disability to walk, and developmental delay from his infancy. He had a four-time history of hospital admissions because of sinusitis, pneumonia, fever, and diarrhea. At the age of 2.5 years, the patient developed Coombs-positive hemolytic anemia that was treated with blood transfusion, IVIG, and prednisolone. At the age of 6 years, his laboratory investigations revealed neutropenia. Leukopenia and lymphopenia were also noted in his BMA sample. Moreover, patient 2 had a history of eczema and cradle cap. The result of the allergic macroarray test revealed food allergy to eggs, hazelnut, pistachio nut, celery, and flour. The other immunological assessments showed normal serum immunoglobulin (Ig) levels, although the IgM isohemagglutinin titer did not show an efficient response (Table 1); therefore he had received IVIG regularly. The undetectable uric acid level in his serum sample suggested PNP deficiency. Next generation sequencing (NGS) of the whole exome was performed using SureSelect V6 Exome Kit (Illumina, San Diego, CA, USA) and the result showed a novel likely pathogenic missense mutation in exon 5 (c.569G > T) of the *PNP* gene (NM_000270.4) which changes Gly 190 to Val (Figure 1B). This variant has been mentioned as of uncertain significance based on VarSome (Pathogenic computational verdict based on 12 pathogenic predictions from BayesDel_addAF, DANN, DEOGEN2, EIGEN, FATHMM-MKL and 7 more vs 1 benign prediction from PrimateAI). The heterozygosity of this mutation in the patient’s parents was confirmed by Sanger sequencing. Moreover, the result of the TREC assay of the patient showed undetectable levels of TREC, confirming T cell deficiency. He received hematopoietic stem cell transplantation (HSCT) from his grandmother for the treatment and after 6 months, he is in good condition with complete engraftment. Moreover, his allergic reaction has significantly diminished clinically.

## Discussion

Here, we introduce two Iranian patients in individual families with PNP deficiency and no family history of recurrent infections and immunologic disorders. Genetic analyses of these patients revealed a novel deletion in exon 3 and a novel missense mutation in Exon 5 in their *PNP* genes, respectively. The most common manifestation in PNP patients is recurrent infections, mainly respiratory tract infections. Defective mitochondrial function or depletion of dGTP in neurons result in neurologic abnormalities, hypotonia, tremors, retarded motor development, ataxia, and behavioral difficulties. Varying degrees of mental retardation have been reported in more than half of the PNP-deficient patients.^2,8,10^ Both patients in this study presented neurological defects, as well.

The defective transformation of inosine into hypoxanthine is suggestive of PNP deficiency which leads to hypouricemia. PNP deficiency could also be characterized by lymphopenia associated with low levels of uric acid and increased levels of inosine.^7,11^ Patient 1 manifested T cell deficiency, gastroenteritis, and pneumonia from early infancy.

In addition to the above-mentioned symptoms, patient 2 suffered from neutropenia, anemia, eosinophilia, and severe food allergy. Versatile anomalies and symptoms in these patients are related to the vital biological function of the PNP enzyme and purine metabolites.^2^ Fewer than half of PNP deficiency patients develop autoimmune diseases, notably systemic lupus erythematosus, idiopathic thrombocytopenic purpura and autoimmune hemolytic anemia, while both patients in this study suffered from anemia.^12,13^ It has been suggested that PNP deficiency results in increased activation of TLR7 and subsequently IL-6 up-regulation leading to autoimmunity.^14^

Previously, Dror et alreported a patient with PNP deficiency and eosinophilia.^15^ It is assumed that primary immunodeficiency (PID) could be a potential cause of eosinophilia, several primary immunodeficiencies including T cells deficiency, phagocytic dysfunction, and cytokine signaling disorders are reported to be related to eosinophilia; therefore, clinical evaluation of patients with eosinophilia has a considerable impact on early diagnosis of patients who may have immunodeficiencies.^16,17^

Recently, TREC/KREC assay has been proven as a tool for newborn screening to identify patients with primary immunodeficiency diseases including SCID and X-linked agammaglobulinemia (XLA).^18^ Therefore, early diagnosis of this disorder through TREC/KREC assay could be useful in the effective treatment of patients by allogeneic HSCT, enzyme replacement, and gene therapy.^19,20^ The result of the TREC assay of patient 2 showed undetectable levels of TREC, which follows the immunophenotyping findings of this patient. This is in agreement with the previous studies which showed that PNP deficiency could be detected by TREC assay.^21,22^

Previously, a missense *PNP* gene variant has been reported with no obvious effect on PNP activity and also normal uric acid level was found in an affected child with a mutation in the *PNP* gene.^23^ Moreover, developmental delay has been reported in two Iranian patients with PNP.^24^ However, a recent study showed no neurological defects and developmental delay in an Iranian PNP-deficient patient along with IgA deficiency.^25^ Therefore, a definite diagnosis of the disease is based on genetic analysis and measuring PNP activity. The investigated mutations in the current study are likely pathogenic based on the American College of Medical Genetics and Genomics (ACMG) guideline.^26^

PNP is a homotrimer protein and each monomer consist of 295 amino acids. The purine binding site is composed of Ala116, Phe200, Glu201, Val217, Met219, Thr242, and Asn243 residues.^27^ Besides, each monomer has 4 regulatory phosphate binding sites and 3 sulfate ligand binding sites. Patient 1 in this study had a large deletion Gly83_Lys95 + 9 bp intron in exon 3 which includes Arg84, Phe85, and His86 that comprise a phosphate binding site; Arg84 and His86 are also involved in sulfate binding site.^28^ This deletion extended through the splice site and aberrant splicing that leads to intron retention or exon skipping (Figure 1A, B). The second mutation in patient 2 changes the conserved glycine190 (an amino acid without a side chain) to valine which may change protein conformation and function, as well (Figure 1C).

 It is notable to mention that no allele frequencies were reported in the 1000 genome project for these variations. We must acknowledge the limitations of our study when discussing the enzyme activity of PNP and the expression of its protein.

## Conclusion

 Due to the poor prognosis of PNP deficiency, early diagnosis and proper treatment are vital for these patients. Currently, HSCT is the only available cure; hopefully, other efficient treatments, notably gene therapy or enzyme replacement, will be developed for PNP-deficient patients.
